# Bacteria of the Genus *Xenorhabdus*, a Novel Source of Bioactive Compounds

**DOI:** 10.3389/fmicb.2018.03177

**Published:** 2018-12-19

**Authors:** Jönike Dreyer, Antoinette P. Malan, Leon M. T. Dicks

**Affiliations:** ^1^Department of Microbiology, Stellenbosch University, Stellenbosch, South Africa; ^2^Department of Conservation Ecology and Entomology, Stellenbosch University, Stellenbosch, South Africa

**Keywords:** *Xenorhabdus*, bioactive compounds, secondary metabolites, antimicrobial properties, antimicrobial peptides

## Abstract

The genus *Xenorhabdus* of the family Enterobacteriaceae, are mutualistically associated with entomopathogenic nematodes of the genus *Steinernema.* Although most of the associations are species-specific, a specific *Xenorhabdus* sp. may infect more than one *Steinernema* sp. During the *Xenorhabdus–Steinernema* life cycle, insect larvae are infected and killed, while both mutualists produce bioactive compounds. These compounds act synergistically to ensure reproduction and proliferation of the nematodes and bacteria. A single strain of *Xenorhabdus* may produce a variety of antibacterial and antifungal compounds, some of which are also active against insects, nematodes, protozoa, and cancer cells. Antimicrobial compounds produced by *Xenorhabdus* spp. have not been researched to the same extent as other soil bacteria and they may hold the answer to novel antibacterial and antifungal compounds. This review summarizes the bioactive secondary metabolites produced by *Xenorhabdus* spp. and their application in disease control. Gene regulation and increasing the production of a few of these antimicrobial compounds are discussed. Aspects limiting future development of these novel bioactive compounds are also pointed out.

## Introduction

Since the discovery of penicillin in 1928 and the introduction of sulphonamides in 1935, more than 20 classes of antibiotics entered the market (Coates et al., 2002; Powers, 2004). The majority of these antibiotics were developed between 1940 and 1962 (Coates et al., 2011). No novel classes of antibiotics were developed between 1968 and 1998. Antibiotics developed up to 1960 protected humans from infections for approximately 50 years (Coates et al., 2011). Within 2 years of marketing, resistance is usually observed, even to new classes of compounds (Bax et al., 1998). With the current rate at which bacteria develop resistance, we may need more than 20 new classes of antibiotics to last us for the next 50 years (thus up to 2060).

Infections caused by Gram-negative bacteria are difficult to treat and many produce metallo-β-lactamase that neutralize carbapenems (Kumarasamy et al., 2010). Strains of *Staphylococcus aureus* developed resistance to penicillin in the early 1940s, shortly after its introduction into the market (Chambers and Deleo, 2009). Resistance to methicillin was recorded just 1 year after its introduction. Nowadays, most of the deaths caused by *S. aureus* are due to MRSA (Klein et al., 2007). Other pathogens in the so-called ESKAPE group for which new antibiotics are urgently needed are *Enterococcus faecium, Acinetobacter baumanii, Klebsiella pneumoniae, Pseudomonas aeruginosa*, and *Enterobacter* species (Rice, 2010).

Another emerging pathogen is *Clostridium difficile.* Infections caused by *C. difficile* increased dramatically over the last decade, especially in patients with irritable bowel disease (IBD) (Nguyen et al., 2008). Severe cases of CDI are treated with oral metronidazole (250–500 mg four times a day for 10–14 days), or oral vancomycin (125–500 mg four times a day for 10–14 days). Metronidazole is often administered intravenously, in doses of 500 mg four times daily (Persky and Brandt, 2000). Although metronidazole is the antibiotic of choice, failure rates of 22 to 38% have been reported and many strains have developed resistance (Miller et al., 2010, 2011).

The rate at which bacteria develop resistance to antibiotics depends on the genetic characteristics of the pathogen and the mode of action of the antibiotic. For instance, resistance to rifampicin, which inhibits DNA-dependent RNA polymerase, develops much faster than resistance to antibiotics that target cell membranes (Lambert, 2005; Zhanel et al., 2008). Bacteria have several mechanisms to protect themselves against antibiotics, e.g., (i) inactivation of the antibiotic (e.g., production of β-lactamase degrading the β-lactam ring in penicillins and cephalosporins), (ii) changing membrane permeability to reduce the uptake of an antibiotic, (iii) changing efflux pumps to increase the excretion of an antibiotic from the cell, (iv) increasing production of the target enzyme, (v) finding alternative mechanisms to bypass damaged cell components, and (vi) altering the target site to render the antibiotic ineffective. Some antibiotics, such as fluoroquinolones, induce the SOS response in cells, which increases the level of errors in DNA replication and by doing so, increases resistance (Da Re et al., 2009).

It is evident that we need novel antibiotics to treat bacterial infections. Oxazolidinone (linezolid by Pfizer) and cyclic lipopeptide (daptomycin by Cubist), with activity against Gram-positive bacteria, including MRSA, are two of the most recent antibiotics released into the market (Coates et al., 2011). There may be a number of yet to be published antibiotics that are currently in preclinical development, but the overall conclusion is that we are heading for a disaster if antibiotics with broader antimicrobial activity are not developed in the next few years. The rate at which novel antibiotics are being developed is just not sufficient to control bacterial infections. We need to focus our efforts in developing antibiotics that target complex bacterial systems, such as cell membranes.

*Xenorhabdus* spp. produce various bioactive compounds throughout their life cycle and the genus is an underestimated and neglected source of novel bioactive compounds. All strains live in close association with entomopathogenic nematodes (EPNs) of the family Steinernematidae. Biologically active compounds produced by *Xenorhabdus* spp. have a broad-spectrum of antimicrobial activity, inhibiting the growth of bacteria, fungi and protozoa, the development of insects and nematodes, and the formation of cancerous cells (Webster et al., 2002). The variety of bioactive compounds produced by *Xenorhabdus* spp. differ, even between strains of the same species. Polyketide synthetases (PKS) and non-ribosomal peptide synthetases (NRPS) are responsible for the production of a diverse group of peptides, e.g., depsipeptides (Lang et al., 2008; Zhou et al., 2013; Kronenwerth et al., 2014), xenocoumacins (Reimer, 2013), and PAX (peptide-antimicrobial-*Xenorhabdus*) peptides (Fuchs et al., 2011). Other *Xenorhabdus* antimicrobial compounds include benzylideneacetone (Ji et al., 2004), indole derivatives (Sundar and Chang, 1993), and ribosomal-encoded bacteriocins (Thaler et al., 1995; Singh and Banerjee, 2008).

**FIGURE 1:**
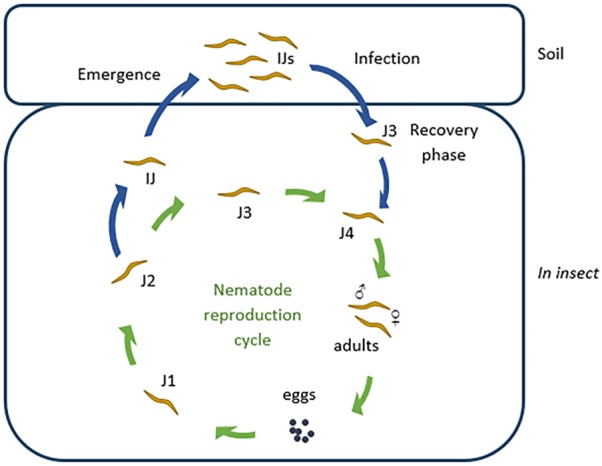
The *Steinernema* life cycle. The infective juvenile (IJ) nematodes infect an insect host and recover to the feeding phase (J3). J3 nematodes molt into fourth phase (J4) juveniles, which in turn develop into male and female adults. These adults reproduce and lay eggs. The eggs hatch as first phase juveniles (J1) which feed and molt to second, third, and fourth juvenile phases (J2–J4), and ultimately into adults. After one to three generations, when nutrients are depleted, second phase juveniles develop into IJs (special third phase juveniles). Each of the IJs host *Xenorhabdus* bacteria in their receptacle. These IJs then leave the cadaver and await a new prey.

The *Steinernema* life cycle is shown in Figure 1. Phase I cells of *Xenorhabdus* are associated with reproducing nematodes, but changes to phase II when nematodes infect the insect cadaver (Akhurst, 1980). Phase I cells are larger than phase II cells, have crystalline inclusion bodies, are mobile (by swarming), and produce proteases, lipases, and bioactive compounds (Akhurst, 1982; Boemare and Akhurst, 1988). The two phases are clearly distinguished by staining with bromothymol blue and triphenyltetrazolium chloride (Kaya and Stock, 1997). Phase I cells streaked onto growth media supplemented with a combination of the two dyes form dark blue colonies with a red core and are surrounded by a clear zone. Exceptions to the rule have been reported, i.e., phase I cells not absorbing bromothymol blue (Koppenhöfer, 2007). Nematodes infected with *Xenorhabdus* enter the insect host through natural openings, such as the mouth, anus and respiratory spiracles, and migrates to the hemocoel. Once in the hemocoel, the nematodes enter a feeding phase, whereupon the bacteria are released (by defecation) and start to produce compounds to repress the insect’s immune system (Webster et al., 2002). The protein UnA, produced by some strains of *Xenorhabdus nematophila*, prevents the hemocytes of the insect to aggregate and form capsules or nodules that would surround the nematodes and bacteria (Ribeiro et al., 1999). Outer membrane proteins and lipopolysaccharides produced by *X. nematophila* prevents adhesion to hemocytes of *Galleria mellonella* Linnaeus (Dunphy and Webster, 1991). This inhibits the activation of phenoloxidase, an important enzyme in the insects’ immune response artillery (Forst et al., 1997). Some strains of *X. nematophila* inhibits the activity of phospholipase A_2_ (PLA_2_), which is partly responsible for the biosynthesis of eicosanoids (Dunphy and Webster, 1991; Park and Kim, 2000, 2003). The absence of eicosanoids results in severe immune depression and causes septicemia. *Xenorhabdus budapestensis* D43 produces a 57 kDa insecticidal protein that activates the phenoloxidase cascade and elicits an intense immune response in *G. mellonella* larvae (Yang et al., 2012). This leads to an excessive production of quinones, which are toxic to the larvae. *X. nematophila, Xenorhabdus japonica, Xenorhabdus kozodoii*, and *Xenorhabdus beddingii* cause apoptosis of insect hemocytes (Dunphy and Webster, 1984; Brillard et al., 2001; Cho and Kim, 2004). In the case of *X. nematophila*, the compound responsible for cytotoxicity has been identified as protein CyA. Production of these exoenzymes and toxins occur hours, killing the insect within 24 to 48 h (Khush and Lemaitre, 2000). With the depletion of nutrients, the juvenile nematodes switch to the infective juvenile (IJ) stage and re-associates with the bacteria. The nematodes then leave the cadaver in search of a new host (Poinar, 1990; Goodrich-Blair and Clarke, 2007; Stock and Goodrich-Blair, 2008). The genomes of *Xenorhabdus* spp. are large (e.g., 4.43 Mb in the case of *X. nematophila*) and the cells often harbor megaplasmids (e.g., 155 kba). Recent studies have shown that the genomic DNA of *Xenorhabdus* spp. contain many genes involved in the synthesis of insecticidal and antimicrobial compounds (Ogier et al., 2010; Chaston et al., 2011). Recent findings of gene rearrangements suggests that *Xenorhabdus* spp. are highly adaptable to environmental changes (Ogier et al., 2010).

Until recently, the general assumption was that a specific *Xenorhabdus* sp. can only infect one *Steinernema* species. Sicard et al. (2004) has shown that the fitness of *Steinernema carpocapsae* improved when associated with *X. nematophila*, but not when associated with non-native *Xenorhabdus* spp. Murfin et al. (2015) reported an increase in the fitness of *Steinernema* nematodes when infected with a strain of *Xenorhabdus bovienii* native to the nematode, or when associated with a strain from another *Steinernema* sp. closely related to the original nematode. Some authors hypothesized that the association of a specific *Xenorhabdus* sp. with more than one *Steinernema* sp. is an indication that the respective nematodes are phylogenetically related. The findings of Lee and Stock (2010) provided the final answer to this hypothesis by showing that host switching of *Xenorhabdus* spp. may occur within clades and between clades, up to 17 times. *Steinernema beitlechemie* from the *Cameroonense-*clade (Çimen et al., 2016) is associated with *Xenorhabdus khoisanae.* However, *X. khoisanae* was first isolated from *Steinernema khoisanae* of the *Glaseri*-clade (Nguyen et al., 2006; Ferreira et al., 2013). In a more recent paper, further evidence of *X. khoisanae* switching between clades was reported when the species was isolated from *Steinernema sacchari* of the *Cameroonense-*clade (Dreyer et al., 2017).

*Steinernema* nematodes infected with *Xenorhabdus* spp. is a highly effective natural way of controlling insect pests. *Steinernema yirgalemense* infected with a few as 50 infected juvenile nematodes (IJs) caused 100% mortality of false codling moth (*Thaumatotibia leucotreta*, Meyrick) larvae (Malan et al., 2011). Other studies have shown similar results against mealy bugs (*Planococcus ficus*, Signoret) (Le Vieux and Malan, 2013), sugarcane stalk borer (*Eldana saccharina*, Walker) (Pillay et al., 2009), fruit flies *Ceratitis capitate* (Wiedemann) and *Ceratitis rosa* (Karsch) (Malan and Manrakhan, 2009; James et al., 2018).

## Secondary Metabolites Produced by *Xenorhabdus* Species

*Xenorhabdus* bacteria are known to produce broad-spectrum compounds with activity against bacteria, fungi, insects, nematodes, protozoa, and cancer cells (Webster et al., 2002). These activities each play a unique role in the protection and bioconversion of the host cadaver, and promote reproduction and growth of the nematodes. Paul et al. (1981) identified several novel antibacterial compounds produced by *Xenorhabdus* spp. Since this discovery, various additional bioactive *Xenorhabdus* compounds have been reported. Fodor et al. (2012) summarized novel antimicrobial peptides produced by *Xenorhabdus szentirmaii* and *X. budapestensis*, and focused mostly on the effect these peptides have on the plant pathogens *Agrobacterium, Burkholderia, Clavibacter, Curtobacterium, Dyckeya, Erwinia, Pectobacterium, Ralstonia, Pseudomonas, Xanthomonas, Phytophthora, Pythium, Botrytis, Alternaria*, and *Fusarium* species.

Only a few papers have been published on the regulation of antimicrobial compounds produced by *Xenorhabdus* spp. In *X. nematophila*, the leucine responsive protein (lrp) plays a role in regulating symbiosis with nematodes and pathogenicity to insects (Cowles et al., 2007; Hussa et al., 2015). Lrp may also be involved in regulating antibiotic production, as strains without the *lrp* gene had no antimicrobial activity toward *Micrococcus luteus* and *Bacillus subtilis* (Cowles et al., 2007). In the case of *Photorhabdus luminescens*, lrp led to the overproduction of desmethylphurealipid A. Injection of desmethylphurealipid A into *G. mellonella* and *Manduca sexta* larvae reduced the mRNA levels for antimicrobial-peptide-encoding genes, suggesting that these molecules may play a role in insect pathogenicity (Nollmann et al., 2015). Based on results obtained with microarray analyses (Engel et al., 2017), lrp regulates genes encoding the biosynthesis of xenematides, xenortides, rhabdopeptides, xenocoumacins and PAX-peptides, as well as six other genes with an unknown biosynthetic function. In *Salmonella enterica*, the LysR-type transcriptional regulator protein (LeuO) acts as a repressor for SPI-1 (*Salmonella* pathogenicity island) and is often described as an antagonist of heat-stable nucleoid-structuring protein (H-NS) (Chen and Wu, 2005; Espinosa and Casadesús, 2014). In *Vibrio cholerae*, LeuO is part of the ToxR (a membrane-spanning transcription factor) regulon and down-regulates important virulence factors. In *X. nematophila* and *P. luminescens* LeuO is described as a regulator for virulence factors and plays a role in the regulation of natural products. LeuO attenuates the production of most examined natural products, including nematophin, an antifungal and antibacterial peptide (Li et al., 1997a). Xenocoumacins, xenematides, and xenortides display antimicrobial activity against a broad spectrum of bacteria (Lang et al., 2008) and help eliminate microorganisms from the insect cadaver. Xenematides have insecticidal activity, rhabdopeptides are active against insect hemocytes (Lang et al., 2008; Reimer et al., 2013), and xenortides are cytotoxic against mammalian L6 cells (Reimer et al., 2014). These antimicrobial compounds are often produced at very low concentrations. Production levels may be scaled up by exchanging the promoters in front of biosynthesis genes with stronger or constitutive promoters (Bode et al., 2015), clone the genes into a replicative overexpression plasmid, or express the biosynthesis genes in different hosts (Schimming et al., 2014). Engel et al. (2017) reported that expression of the LeuO regulator of *X. nematophila* in *X. szentirmaii* led to overproduction of the GameXPeptide. The authors have also shown that this may lead to the production of antimicrobial compounds normally not produced by the natural host.

**FIGURE 2:**
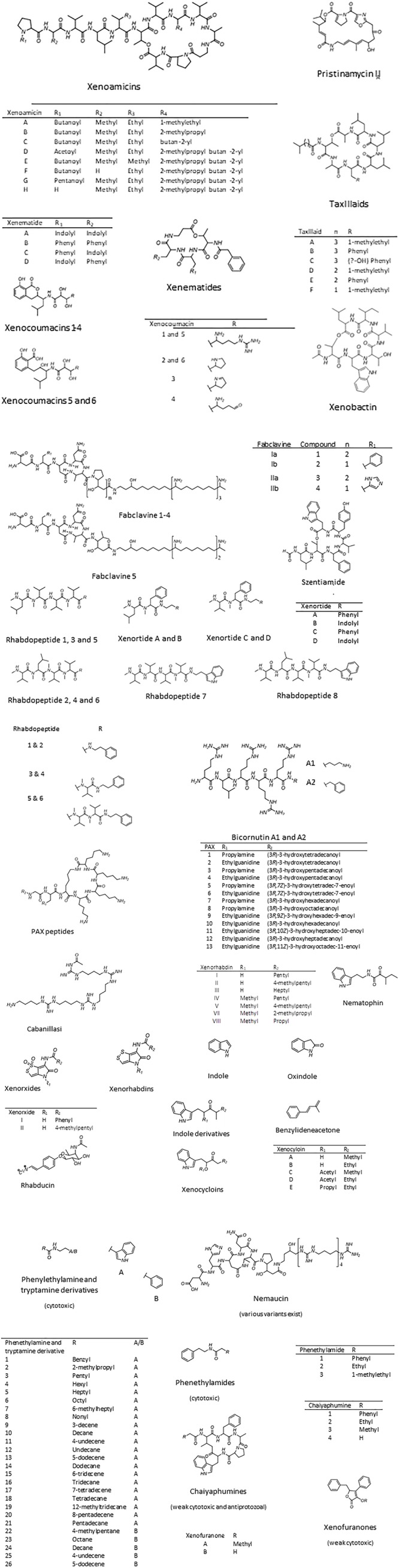
*Xenorhabdus* bioactive compounds. Bioactive compounds with unknown structures include the antibacterial xenoprotec, bicornituns C and D, and the two bacteriocins, xenorhabdicin, and xenocin. Compiled from Boemare et al. (1992), Paik et al. (2001), Hwang et al. (2003), El-Hag and El-Sadawy (2008), Gaultieri et al. (2012).

Several classes of structurally diverse secondary metabolites with a broad spectrum of bioactivity, including insecticidal, antifungal, antibacterial, nematicidal, and cytotoxicity, have been isolated from different *Xenorhabdus* strains (Brachmann and Bode, 2013). These include depsipeptides such as xenematides, xenocoumacins, fabclavines, pristinamycin, xenortides, rhabdopeptides, bicornitun, PAX peptides, cabanillasin, nemaucin, dithiolopyrrolone derivatives, indole-containing compounds, benzylideneacetone, rhabduscin, bacteriocins and a few unnamed peptides (Table 1). The diverse profile of bioactive compounds produced by *Xenorhabdus* spp. is supported by the large variation in gene clusters (as many as 23; Reimer et al., 2014). The secondary metabolic compounds produced by the *Xenorhabdus* spp. included in this review, and the accession numbers of the respective genome and gene sequences listed in GenBank, are included in Table 1. The molecular structures of the bioactive compounds are shown in Figure 2. Despite the large variation in bioactive compounds, none have been commercialized in chemical form. However, several applications of nematodes associated with *Xenorhabdus* symbionts have been used as biological control agents (Ehlers, 2001; Chavarría-Hernández et al., 2010).

**Table 1:** Genome and gene assemblies of *Xenorhabdus* spp. and production of secondary antimicrobial compounds.

Species	DNA assembly	Secondary metabolic compound described	Reference
*X. beddingii*	MUBK00000000	R-type bacteriocins	Akhurst, 1986a,b; Boemare et al., 1992
*X. bovienii*	FN667741	Amicoumacin, xenomin, xenorxid, xenorhabdin, xenematide	Chaston et al., 2011; Sugar et al., 2012; Park et al., 2016
*X. budapestensis*	NIBS01000000	Fabclavine, bicornitun, unnamed peptide	Böszörményi et al., 2009; Xiao et al., 2012; Fuchs et al., 2014; Tobias et al., 2017
*X. cabanillasii*	NJGH01000000 CBXE00000000	Nemaucin, cabanillasin, rhabdopeptide	Gualtieri et al., 2009; Houard et al., 2013; Reimer et al., 2013
*X*. *doucetiae*	FO704550, FO704549	Xenoamicin, xenocoumacin, xenorhabdin	Zhou et al., 2013; Bode et al., 2017
		phenylethylamine, tryptamide	
*X*. *indica*		Lipodepsipeptide	Kronenwerth et al., 2014
*X*. *japonica*	FOVO00000000		Nishimura et al., 1994
*X*. *khoisanae*	LFCV01000000		Crawford et al., 2012
*X*. *kozodoii*	NJCX01000000	Xenocoumacin	Reimer et al., 2009; Tobias et al., 2017
*X*. *mauleonii*	NITY01000000 FORG00000000	Xenoamicin, xenocoumacin, xenorhabdin	Zhou et al., 2013; Tobias et al., 2017
*X. nematophila*	FN667742, FN667743, LN681227, LN681228, JRJV00000000, CCWW00000000,	Rhabduscin (putative gene cluster sequenced)	Chaston et al., 2011; Crawford et al., 2012; Lanois et al., 2013; Hong et al., 2015
	CCWM00000000, CAVM00000000	Pristinamycin	Brachmann et al., 2012; Shi and Bode, 2018
		Xenocoumacin (gene cluster *xcnA*–*xcnN* sequenced)	Mclnerney et al., 1991a; Lang et al., 2008; Park et al., 2009; Reimer et al., 2009; Guo et al., 2017
		Xenematide	Lang et al., 2008; Crawford et al., 2011
		Rhabdopeptide (peptides rdpA, B, and C sequenced)	Reimer et al., 2013
		PAX peptide (peptides xpsA, xpsB, and xpsD sequenced)	Gualtieri et al., 2009; Crawford et al., 2010; Fuchs et al., 2011
		Oxindole and benzylideneacetone	Seo et al., 2012
		Nematophin	Li et al., 1997a,b; Lang et al., 2008; Crawford et al., 2011; Reimer et al., 2014
		Xenortide (peptides NRPS XndA and B sequenced)	Lang et al., 2008
		Xenorhabdicin	Thaler et al., 1995; Morales-Soto and Forst, 2011; Morales-Soto et al., 2012
		Xenocin (peptide xcinA sequenced)	Singh and Banerjee, 2008
*X. szentirmaii*	Genome assembly NIUA01000000 NIBV01000000 CBXF00000000	Fabclavine, szentiamide	Fodor et al., 2012 Nollmann et al., 2012; Grundmann et al., 2013; Fuchs et al., 2014; Tobias et al., 2017

*References are key papers on species. For DNA sequences, refer to GenBank. Genome accession numbers were obtained from GenBank [as listed in the PATRIC database, https://www.patricbrc.org/view/Taxonomy/626#view_tab=genomes&filter=eq(genome_status,^∗^)]. Underlined accession numbers: complete genome sequence. Peptide sequences deposited are listed.*

### Depsipeptides

Depsipeptides are peptides with one or more amide group replaced by a hydroxy acid, leading to the formation of an ester bond. These peptides generally contain alternating peptide and ester bonds. Thus far, five classes of depsipeptides have been described. The first class, classified as xenoamicin, are tridecadepsipeptides with hydrophobic amino acids and are produced by *Xenorhabdus doucetiae* and *Xenorhabdus mauleonii* (Zhou et al., 2013). Concluded from the genome sequence of *X. doucetiae* DSM 17909, xenoamicins are encoded by five non-ribosomal peptide synthetases (NRPSs), XabABCD and an aspartic acid decarboxylase (XabE). Thirteen modules within XabABCD have been linked to the synthesis of xenoamicin. XabE is involved in the formation of β-alanine. Based on the hydrophobic characteristics, xenoamicin interacts with the cytoplasmic membrane. However, no antibacterial or antifungal activity has been recorded for xenoamicin A, which implies a different mode of activity. Anti-protozoal and weak cytotoxic activities have been reported for xenoamicin A, but the target sites have not been identified.

The second class of depsipeptides, the lipodepsipeptides, has an additional fatty acid chain attached to one of the amino acids and is produced by *Xenorhabdus indica* (Kronenwerth et al., 2014). The peptides are named after their amino acid sequence, T-A-X-L-L-L-A (X = L, F, or Y), and are referred to as taxlllaids (A-G). Seven variants were described, each classified based on the length of the fatty acid chain, the third amino acid and the overall structure of the molecule, i.e., an open chain or ring structure. The synthesis of taxlllaids are encoded by a gene cluster consisting of two NRPSs, and TxlA and TxlB, with four and three modules, respectively. Natural taxlllaid A and synthetic taxlllaids B-G have antiprotozoal activity. Taxlllaid A is also cytotoxic to human carcinoma cells (HeLa) (Kronenwerth et al., 2014).

The third class of depsipeptides are classified as indole-containing xenematides. Xenematide A was the first example, isolated from *X. nematophila* (Lang et al., 2008). The molecule is cyclic, antibacterial and weakly insecticidal. Three years later, Crawford et al. (2011) isolated three more xenematides (B–D) from *X. nematophila* and showed with gene knockout studies that XNC1_2713 encodes the production of xenematide A. Xenematides are not restricted to *X. nematophila* or the genus *Xenorhabdus*, as protein homologs have been identified in *X. bovienii* and *Photorhabdus asymbiotica.*

The final two depsipeptide classes consist of xenobactin and szentiamide (Nollmann et al., 2012; Grundmann et al., 2013). Xenobactin was isolated from an unknown *Xenorhabdus* sp., strain PB30.3, and szentiamide from *X. szentirmaii.* Both peptides are active against *Plasmodium falciparum* and have some activity against *Trypanosoma brucei rhodesiense* and *Trypanosoma cruzi.* Szentiamide does not have any antibacterial or antifungal activity, but has a weak cytotoxic activity against *G. mellonella* hemocytes. Contrary to szentiamide, xenobactin has no cytotoxic activity, but is active against *M. luteus.* The antibacterial activity is likely due to the hydrophobic nature of the peptide and the target in *M. luteus* is most probably the cell membrane.

### Xenocoumacins

Xenocoumacins, first described by Mclnerney et al. (1991a), have benzopyran in the amino acid chain and are mostly produced by *X. nematophila.* Xcn1 is active against Gram-positive and Gram-negative bacteria, and has antifungal and anti-ulcer activity. Xcn2 has less antibacterial activity and no antifungal activity, but has anti-ulcer activity. More recently, Reimer (2013) discovered that Xcn2 is produced from Xcn1 through reactions encoded by genes *xcnM* and *xcnN.* In a study conducted by Park et al. (2009), the *xcnM* gene was inactivated, which led to an increased Xcn1 level, as expected, but also decreased cell viability 20-fold. The conversion of Xcn1 to Xcn2 was therefore suggested to be a mechanism used by the bacteria to avoid self-toxicity. Xcn1 is modified by various reactions to produce Xcn2, Xcn3, Xcn4, Xcn5, and Xcn6. The latter four were isolated from *X. nematophila* and *X. kozodoii* (Reimer et al., 2009).

### Fabclavines

Fabclavines, or peptide-polyketide-polyamino peptides, are produced by *X. budapestensis* and *X. szentirmaii* and are encoded by a combined PKS-NRPS gene cluster (Fuchs et al., 2014). The peptide moiety is synthesized by the FclI and FclJ NRPSs, while in the PKS, FclK is responsible for catalyzing the elongation of the peptide moiety’s proline residue. These peptides are active against Gram-positive and Gram-negative bacteria, *Saccharomyces cerevisiae, Plasmodium falciparum, Trypanosoma brucei*, and *Trypanosoma cruzi*. Fabclavines and cationic antimicrobial peptides are structurally very similar. The latter displayed increased activity when combined with antibiotics (Hancock, 2001).

### Pristinamycin

Pristinamycin forms part of the streptogramin A family of antibiotics and was until recently known to be produced by streptomycetes only. Pristinamycin consists of approximately 30% pristinamycin I and 70% pristinamycin II. Component II occurs in two forms, pristinamycin II_A_ and II_B_, of which II_A_ is the most abundant (Blanc et al., 1995). Pristinamycin II_A_ is also produced by *X. nematophila* and is encoded by PKS/NRPS (Brachmann et al., 2012). The biosynthetic gene clusters in *X. nematophila* and *Streptomyces pristinaspiralis* are very similar. Interestingly, further analysis of *X. nematophila* showed that it does not contain a gene cluster for the biosynthesis of pristinamycin I_A_. The *pxn* (pristinamycin II_A,_ *X. nematophila*) gene cluster, however, is associated with transposases, suggesting that the genes were obtained through horizontal gene transfer. This might explain the absence of the pristinamycin I_A_ gene cluster in *X. nematophila.*

### Xenortides

To date, four xenortides, namely xenortides A–D, have been identified from *X. nematophila* (Lang et al., 2008; Crawford et al., 2011; Reimer et al., 2014). These peptides are biosynthesized by a gene cluster consisting of two NRPS genes (*xndA* and *xndB*). Xenortides have weak antiprotozoal activity, with the tryptamides (xenortides B and D) being more active than the phenylethylamides (xenortides A and C), and xenortide B being the most active (Reimer et al., 2014).

### Rhabdopeptides

Rhabdopeptides are linear, non-ribosomally produced, and structurally similar to xenortides. A total of eight rhabdopeptides have been identified, rhabdopeptides 1, 2, 3, and 4 are from *X. nematophila*, and 7 and 8 from *Xenorhabdus cabanillasii* (Reimer et al., 2013). Rhabdopeptide 2 has weak cytotoxic activity against myoblasts. Rhabdopeptides 2, 7, and 8 have antiprotozoal activity, and 7 and 8 are weakly hemotoxic. These peptides are produced at high concentrations 4 days after infection, but levels stagnate after 10 days, suggesting that they are important during the insect bioconversion and nematode reproduction stages. The gene cluster responsible for the biosynthesis of these peptides consists of a three-module NRPS gene, RdpABC.

### Bicornitun

*Xenorhabdus budapestensis* produce the arginine rich, bioactive compounds bicornitun A1, A2, B, and C (Böszörményi et al., 2009). The NRPS responsible for the production of bicornitun A1 was identified as BicA. This was determined by cloning the *bicA* gene, which encodes BicA, into an expression vector and expressing bicornitun A1 in *Escherichia coli* (Fuchs et al., 2012). The bicornitun complex (a combination of bicornitun A–C) is cytotoxic toward *Phytophthora nicotianae* by inhibiting colony formation, as well as mycelial growth. *Erwinia amylovora* and *Bacillus subtilis* is also susceptible to the bicornitun complex.

### PAX Peptides

PAX peptides 1 to 5 were first identified by Gualtieri et al. (2009) and described as lysine-rich cyclolipopeptides. These peptides, produced by *X. nematophila*, have antifungal and antibacterial activity. However, they do not show cytotoxic activity and did not lead to increased mortality when injected into insects. An additional eight PAX peptides were identified and their structures elucidated by Fuchs et al. (2011). Three NRPS genes (*paxABC*) are responsible for the biosynthesis of the PAX compounds. The three NRPSs (PaxA, PaxB, and PaxC) contains 3, 9, and 10 domains, respectively.

### Cabanillasin and Nemaucin

The peptides cabanillasin and nemaucin were isolated from *X. cabanillasii.* Cabanillasin was efficient at inhibiting the growth of human pathogenic filamentous fungi and yeasts (Houard et al., 2013). Nemaucin was, active against methicillin resistant *S. aureus* (MRSA). Common genes may be involved in the production of these two peptides, as both have four units of the amino-1 guanidino-butane moiety and are produced by the same organism. Nemaucin is, however, structurally more similar to fabclavine 1a from *X. budapestensis* than cabanillasin, and differs only by having a shorter C-terminal (Fuchs et al., 2014).

### Dithiolopyrrolone Derivatives

These derivatives include the two metabolites, xenorhabdins and xenorxides. Xenorhabdins have a typical heterobicyclic pyrrolinonodithiole core, which is characteristic of dithiolopyrrolone compounds (Challinor and Bode, 2015). Xenorxides, in turn, are structurally similar to xenorhabdins and are produced when the sulfur moiety of xenorhabdins is oxidized (Webster et al., 1996). Xenorhabdins and xenorxides have antibacterial, antifungal and insecticidal activities (Mclnerney et al., 1991a,b; Li et al., 1995). Some of these dithiopyrrolone derivatives have anticancer properties. The suggested mode of action is inhibition of RNA synthesis (Joshi et al., 1982; Oliva et al., 2001).

### Indole-Containing Compounds

Indole is an aromatic heterocyclic compound, consisting of a fused pyrrole- and benzene ring (Sainsbury, 2001). Various bacterial species produce indole and indole derivatives that play a role in the regulation of bacterial physiology (Lee and Lee, 2010). Indole derivatives isolated form *X. nematophila* and *X. bovienii* are active against Gram-positive and Gram-negative bacteria, as well as fungi. Sundar and Chang (1993) studied these compounds and found the mode of action the inhibition of RNA synthesis. Growing bacteria have a relatively narrow range of ppGpp concentrations and indole derivatives increase this concentration, leading to a reduction in RNA synthesis and, ultimately, a reduction in growth rate. Seo et al. (2012) identified the indole-containing compound, oxindole, as well as indole, also produced by *X. nematophila*. These compounds have weak phospholipase A_2_ inhibitory effects. As mentioned elsewhere, phospolipase A_2_ is an enzyme required for the production of eicosanoids. Eicosanoids, in turn, are crucial for activating an immune response in the insect by modulating and mediating hemocyte behavior (Shrestha and Kim, 2007). It is thus safe to assume that indole-containing compounds inhibit the immune response of the insect, making it more susceptible to microbial infection. Proschak et al. (2014) identified additional indole derivatives, xenocycloins (A-E), also produced by *X. bovienii.* These compounds have no antibacterial activities, but xenocycloins B and D are active against *G. mellonella* hemocytes. Xenocyloins therefore, also contribute to the insecticidal activity of these bacteria. Xenematides, discussed under depsipeptides, are also known to contain the indole structure.

Another indole containing compound, nematophin, is highly active against MRSA strains (Li et al., 1997a). In a study conducted by Li et al. (1997b), minimal inhibitory concentrations of nematophin and its derivatives against *S. aureus* strains were determined and it was proven that compounds with an α-carbonyl acyl group inhibited the growth of *S. aureus.* However, compounds where the α-carbonyl acyl group was reduced or transferred to a corresponding α-methoximino acyl group, bioactivity decreased or disappeared. It was therefore suggested, that this α-carbonyl acyl group is essential for the bioactivity of these compounds.

### Unnamed Peptides

Two antimicrobial peptides, GP-19 and EP-20, have been isolated from *X. budapestensis* (Xiao et al., 2012). These peptides show broad-spectrum antimicrobial activity against fungi and bacteria, but the mode of action is yet to be unraveled. GP-19 has a neutral charge and is proposed to cause a disruptive effect on the membrane by mobilizing to the cell surface and possibly penetrating the membrane. As EP-20 has a negative charge it most likely does not have the same mode of action. This peptide is proposed to have an intracellular effect, by inhibiting cell wall, nucleic acid, and protein synthesis.

### Benzylideneacetone

The moderately hydrophobic compound, benzylideneacetone, isolated from *X. nematophila*, is active against Gram-negative plant pathogenic bacteria. This compound has been used in the industry for various applications, including as a flavoring additive in soaps, cosmetics, detergents and cigarettes, as well as a food additive in candy, gelatin, and puddings. Even though it has been used for some time, it was only discovered in 2004 to have antibacterial activity (Ji et al., 2004). Benzylideneacetone also inhibits phospholipases A_2_, which, as described, results in the inhibition of the immune response of the insect (Seo et al., 2012).

### Rhabduscin

Rhabduscin is an insecticidal tyrosine derivative, produced by *X. nematophila*. The insecticidal activity of this compound is achieved by inhibiting the enzyme phenoloxidase to a low nanomolar-level with an IC_50_ measurement of approximately 64.1 nM. Phenoloxidase is important in the melanization pathway of the insect’s immune system. Inhibition thereof leads to inhibition of one of the primary innate immune responses (Crawford et al., 2012).

### Bacteriocins

*Xenorhabdus* bacteria also produce bacteriocins, for example, xenocin, which is produced by *X. nematophila*. Interestingly, the antibacterial activity of xenocin was only observed when bacterial strains were grown in minimal medium and not in enrichment medium such as Luria or nutrient broth. Xenocin production is triggered by a low iron concentration. The role of iron depletion has been proposed to be linked to an iron repressed protein, which may act as a toxin receptor on sensitive bacterial strains. This bacteriocin is therefore, only produced in the host larva when nutrient concentrations are low and competition intensifies (Singh and Banerjee, 2008). Another bacteriocin, produced by *X. nematophila* as well as *X. bovienii*, the phage tail-like xenorhabdicin, is bactericidal (Thaler et al., 1995; Morales-Soto et al., 2012). *Xenorhabdus* owes its activity against closely related bacteria to these bacteriocins, which are essential for keeping the environment free of other *Xenorhabdus* spp. and its sister genus, *Photorhabdus* spp. *X. beddingii* is also able to produce bacteriocins, however these bacteriocins have not been characterized.

## Upregulating the Production of *Xenorhabdus* Antimicrobials

When producing antibiotics, it is of the utmost importance that the fermentation conditions are optimal to avoid the squandering of time and money. Antibiotic production in *Xenorhabdus* has been optimized at various time periods, mostly by one research group from the Northwest University of Agriculture and Forestry, China. This group focused on antibiotic production by *X. bovienii* YL002 (Fang et al., 2012) and, *X. nematophila* TB (Fang et al., 2010) and YL001 (Wang et al., 2008), while another group focused on a specific *X. nematophila* strain isolated from *S. carpocapsae* BJ (Xiufen et al., 2001). Factors taken into consideration for these studies were the environmental parameters; initial medium pH, temperature, rotary speed, inoculation volume, medium volume in flask, fermentation time, dissolved oxygen levels, and growth media.

As expected, the optimization for specific strains varies. There are, however a few trends in the results of these studies. The optimal fermentation conditions are a pH from 6.0 to 8.24, temperature of 25–32°C, rotary speed of 150–220 rpm, inoculation volume of 4–15%, medium volume of 54–100 ml/250 ml flask and a fermentation time of 54–72 h. The dissolved oxygen level was tested for only *X. nematophila* YL001 and was optimal when it was shifted during fermentation from 70% after the first 18 h to 50% for the remaining 54 h. The optimal growth media was tested for *X. nematophila* TB and *X. bovienii* YL002, however, the ingredients and amount of each ingredient differs for the respective recipes.

Crawford et al. (2010) identified one of the main compounds that leads to increased small metabolite production in *X. nematophila*. *Xenorhabdus* bacteria are known to produce higher concentrations of bioactive compounds when in *G. mellonella* hemolymph than grown *in vitro* (Maxwell et al., 1994). Therefore, it was hypothesized that one or more compounds present in insect hemolymph are responsible for activating the production of bioactive compounds. This led to the selective purification of *G. mellonella* hemolymph, which led to the discovery of proline as the activating signal. Supplementing bacterial cultures with D-proline did not increase the production of bioactive compounds, however, L-proline did. L-proline is thought to be a generic activating signal as it is present in various insect larvae.

The addition of L-proline to bacterial cultures led to an increase in xenematide, three indole derivatives and rhabduscin biosynthesis. Another indole-containing compound that was affected by an increase in L-proline is nematophin. This L-proline increase led to a decrease in the production of nematophin but an increase in its reduced derivative. L-proline therefore, regulates a metabolic shift in this case, rather than an increase in nematophin production.

It is evident that production of bioactive compounds requires optimization of the production protocol. This is necessary both for use in industry, as well as in research. The optimization process is however not an easy task and extended research is needed for this process, especially since the protocol will be specific for each bacterial strain and product desired.

## Conclusion

Even though *Xenorhabdus* is not one of the generally known antimicrobial metabolite sources, it is clear to see why Pidot et al. (2014) refer to it as a neglected antibiotic source. It is evident that *Xenorhabdus* bacteria are an excellent source for novel antimicrobial metabolites. Various studies (Barkai-Golan, 2001; Böszörményi et al., 2009; Shapiro-Ilan et al., 2009; Fang et al., 2011), have revealed the significant potential of these bioactive secondary metabolites not only *in vitro*, but also *in vivo*. These studies investigated the use of these compounds in only the agricultural industry. However, these compounds may also be exploited in various other industries, including the healthcare and food industries.

A number of papers have been published on *Xenorhabdus* bacteria and their bioactive compounds. However, this is only the tip of the iceberg. A study done by Crawford et al. (2010), stated that the *X. nematophila* DSM 3370^T^ genome contains various gene clusters encoding small molecule antimicrobial metabolites. The number of potential metabolites estimated to be produced by this bacterium vastly exceeds the amount of known antibiotic metabolites. Furthermore, it is generally known that different *Xenorhabdus* species, and even strains, produce different bioactive compounds. Therefore, it is clear that the possibilities regarding novel bioactive compounds produced by *Xenorhabdus* bacteria are virtually endless. Furthermore, taking into consideration the current antibiotic resistance crisis, novel antibiotic discovery is of the essence and *Xenorhabdus* bacteria might hold the key to human survival in the 21^st^ century.

Although the possibilities of discovering novel antimicrobial compounds from *Xenorhabdus* spp. is promising, methods need to be developed to produce these compounds at much higher concentrations. This may be difficult, as most of these antimicrobial compounds are produced non-ribosomally and are thus not a single gene product. However, increased production will follow as we gain more insight on the control of the metabolic pathways. Further research is also required on the effect different growth media and nutrients may have on the production of these bioactive compounds.

## Author Contributions

JD and LD wrote the manuscript. AM revised the manuscript.

## Conflict of Interest Statement

The authors declare that the research was conducted in the absence of any commercial or financial relationships that could be construed as a potential conflict of interest.
